# Antibody-Based Correlates of Protection against Cholera: Analysis of a Challenge Study of a Cholera-Naive Population

**DOI:** 10.1128/CVI.00098-17

**Published:** 2017-08-04

**Authors:** Douglas J. Haney, Michael D. Lock, Jakub K. Simon, Jason Harris, Marc Gurwith

**Affiliations:** a PaxVax, Inc., Redwood City, California, USA; b Division of Infectious Diseases, Massachusetts General Hospital, Boston, Massachusetts, USA; Food and Drug Administration

**Keywords:** bridging, challenge study, cholera, cholera naive, correlate of protection, seroconversion, vaccine, vibriocidal antibodies

## Abstract

Immunologic correlates of protection can be used to infer vaccine efficacy for populations in which challenge trials or field studies are infeasible. In a recent cholera challenge trial (W. H. Chen et al., Clin Infect Dis 62:1329–1335, 2016, https://doi.org/10.1093/cid/ciw145), 134 North American cholera-naive volunteers were randomized to receive either the live, attenuated single-dose cholera vaccine CVD (Center for Vaccine Development) 103-HgR or placebo, and the titers of vibriocidal antibodies against the classical Inaba strain were assessed at 10 days after treatment. Subsequent to the immunologic evaluation, each subject ingested a fixed quantity of virulent Vibrio cholerae O1 El Tor Inaba. Data from this trial suggest that the vaccine-induced increase in the vibriocidal antibody titer prior to challenge is tightly linked with protection: 51/51 vaccinees with postvaccination vibriocidal antibody titers of ≥2,560 were protected against moderate/severe diarrhea, and 60/62 vaccinees who seroconverted or experienced a 4-fold or greater increase in vibriocidal antibody titer relative to prevaccination levels were similarly protected. Atypically high vibriocidal antibody titers were observed in some placebo subjects; protection was limited in these individuals and differed substantially from the level of protection experienced by vaccinees with the same postvaccination titers. Since only 1 of 66 placebo recipients experienced seroconversion, seroconversion was found to be uniquely associated with vaccination and insensitive to the effects of factors that can cause titers to be elevated but are weakly associated with protection. Thus, vibriocidal antibody seroconversion was found to be better than the vibriocidal antibody titer for inferring vaccine efficacy in cholera-naive populations for which studies based upon exposure to V. cholerae are impractical. (This study has been registered at ClinicalTrials.gov under registration no. NCT01895855.)

## INTRODUCTION

The efficacy of a vaccine may be established directly through field studies and challenge trials or may be inferred through correlates of protection. Field studies provide the most naturalistic approach for establishing the efficacy of an experimental vaccine, since they are based upon real-world exposure to the infectious agent. However, the incidence of disease among unvaccinated individuals in field studies is unpredictable and is sometimes too low to demonstrate vaccine efficacy. For certain infectious agents, experimental human challenge trials provide an alternative to field studies. In these challenge trials, volunteers are randomly assigned to receive an experimental vaccine or placebo and are then exposed to a controlled dose of pathogen.

In contrast to the direct methods for establishing vaccine efficacy, the use of correlates of protection comprises an indirect method that is typically based upon vaccine-induced immune responses that are associated with protection against disease. Immunologic correlates of protection may be utilized to infer the efficacy of a vaccine in populations that differ from those in which efficacy was assessed directly or to evaluate new vaccines or formulations in comparison with established vaccines. Correlates may also be useful for inferring protection in individual vaccinees on the basis of their immunologic response to a vaccine or their previous natural exposure to protective antigens.

The oral, live, attenuated Vibrio cholerae O1 classical Inaba strain CVD (Center for Vaccine Development) 103-HgR has been described previously (1). CVD 103-HgR received regulatory approval outside the United States and was used primarily in Switzerland, New Zealand, Australia, and Canada until production was discontinued in 2001. In 2013, as part of a program to redevelop CVD 103-HgR as a vaccine for travelers from the United States to areas where cholera is endemic or epidemic, the efficacy of the vaccine was evaluated in a placebo-controlled challenge study in healthy North American adults between the ages of 18 and 45 years. In this challenge study, the observed protective efficacy of the vaccine was 90% for volunteers challenged at 10 days postvaccination and 79% at 90 days postvaccination (1) (ClinicalTrials registration no. NCT01895855).

CVD 103-HgR has been shown to induce the production of serum vibriocidal antibodies (2). Vibriocidal antibodies are complement-fixing bactericidal antibodies that are primarily directed against the O antigen moiety of the lipopolysaccharide (LPS) antigen (3), and an association between high titers of these antibodies and protection against cholera has been observed for populations living in areas where cholera is endemic (4, 5). Since an effective cholera vaccine must engender a local immune response in the gut mucosa, the vibriocidal antibody titer in serum is considered to be only an indirect marker of an appropriate intestinal response to vaccination, and the vibriocidal response is viewed as a nonmechanistic correlate of protection. The local secretory IgA response in the small intestine might be the most direct predictor of protection, but it is not practical to measure in large studies. Assessment of the postvaccination serum vibriocidal response has thus been postulated to be the most useful approach for inferring the immunologic protectiveness of a cholera vaccine (6).

In addition to eliciting a vibriocidal response, CVD 103-HgR also induces antibodies to the binding B subunit of cholera toxin (CT) (2). The severe and potentially life-threatening disease caused by V. cholerae is a result of active fluid secretion into the gastrointestinal tract lumen induced by CT (7). In previous studies of CVD 103-HgR (2, 8, 9), demonstrably fewer vaccinees generated an anti-CT response than a vibriocidal response, and the anti-CT response has not been proposed to be a strong correlate of protection.

The paper by Chen at al. focused upon the protective efficacy of CVD 103-HgR and also showed that the serum vibriocidal antibody response to vaccination was strongly associated with protection against experimental cholera (1). In this paper, we expand upon the analyses by Chen et al. (1) to identify the best way to utilize the vibriocidal response as a bridge for the inference of efficacy in populations, such as children or older adults, for which participation in volunteer cholera challenge studies might be inappropriate or impractical. These new analyses include a detailed comparison of the value of the postvaccination vibriocidal antibody titer with that of the vaccine-induced increase in the vibriocidal antibody titer relative to prevaccination levels.

Potential correlates of protection were considered to be good candidates for inferring efficacy in a new population if they were strongly associated with the outcome. A candidate was considered to be even more valuable if it was expressed in a high proportion of protected vaccinees. Without this broad-based inclusiveness, a correlate-based rule might be tightly associated with the outcome for a small group of subjects but fail to summarize the full range of protective responses and thus would mirror the vaccine's protective effect for only a portion of a given population.

## RESULTS

### Longitudinal profile of immune response.

In both the cohort challenged at 10 days and the cohort challenged at 90 days, the geometric mean titer (GMT) of vibriocidal antibodies in vaccinated subjects reached its maximum on day 10 following vaccination, and the difference in the vibriocidal response between the 62 protected vaccinees and the 6 unprotected vaccinees was also the greatest on day 10 (Table 1). On the basis of these findings, day 10 postvaccination was selected as the most promising time point at which to assess vibriocidal correlates of protection for immunologic bridging, and the summaries of the vibriocidal antibody results focus on the day 10 data in both challenge cohorts.

**TABLE 1:** Prechallenge vibriocidal antibody GMTs by time point for vaccinees and placebo subjects in each challenge group

Day	GMT							
	Vaccinees challenged at day 10		Vaccinees challenged at day 90		Placebo subjects challenged at day 10		Placebo subjects challenged at day 90	
	Unprotected (*n* = 2)	Protected (*n* = 33)	Unprotected (*n* = 4)	Protected (*n* = 29)	Developed moderate/severe diarrhea (*n* = 20)	Did not develop moderate/severe diarrhea (*n* = 13)	Developed moderate/severe diarrhea (*n* = 19)	Did not develop moderate/severe diarrhea (*n* = 14)
0	40	62	57	34	83	94	35	69
7	40	1,335	95	853	75	110	36	69
10	80	7,793	113	5,244	77	110	37	72
28			67	2,065			35	55
90			67	328			39	66

### Association between candidate correlates of protection and outcome.

Table 2 provides quantitative summaries of the relationships between the total postchallenge stool volume and the vibriocidal antibody titer/fold increase in titer. In the combined group of vaccinees from both challenge cohorts, lower postchallenge stool volumes were associated with higher day 10 vibriocidal antibody titers and with greater fold increases in the vibriocidal antibody titer from day 0 to day 10 (*P* < 0.001 and *P* = 0.004, respectively).

**TABLE 2:** Total stool volume versus day 10 vibriocidal antibody titer and total stool volume versus fold increase in vibriocidal antibody titer from day 0 to day 10 in each treatment group

Assessment	Vaccine group		Placebo group	
	No. of subjects	Mean (median) total stool volume (liters)	No. of subjects	Mean (median) total stool volume (liters)
Day 10 vibriocidal antibody titer				
20–80	5	7.1 (3.9)	46	6.2 (5.4)
160–640	6	2.5 (0.7)	15	3.3 (2.2)
1,280–2,560	11	0.4 (0.2)	4	2.5 (2.2)
5,120–10,240	33	0.1 (0.0)	1	0.1 (0.1)
20,480–81,920	13	0.1 (0.0)	0	
Fold increase in vibriocidal antibody titer from day 0 to day 10				
≤2	6	7.1 (6.8)	65	5.3 (4.4)
4–8	4	2.2 (2.5)	0	
16–32	12	0.1 (0.0)	1	0.0 (0.0)
64–128	18	0.2 (0.0)	0	
256–512	18	0.1 (0.0)	0	
1,024–4,096	10	0.1 (0.0)	0	

Interestingly, even though the cohort was drawn from a cholera-naive population, some subjects had evidence of preexisting serum vibriocidal activity, and in the combined group of placebo subjects from both challenge cohorts, lower postchallenge stool volumes were associated with higher day 0 and day 10 vibriocidal antibody titers (*P* = 0.04 and *P* = 0.02, respectively). Placebo subjects experienced little to no change in vibriocidal antibody titer over time, and the association between the fold increase in titer and the outcome in placebo subjects was not assessed.

### Vaccine-specific vibriocidal responses and implications for bridging.

**FIG 1:**
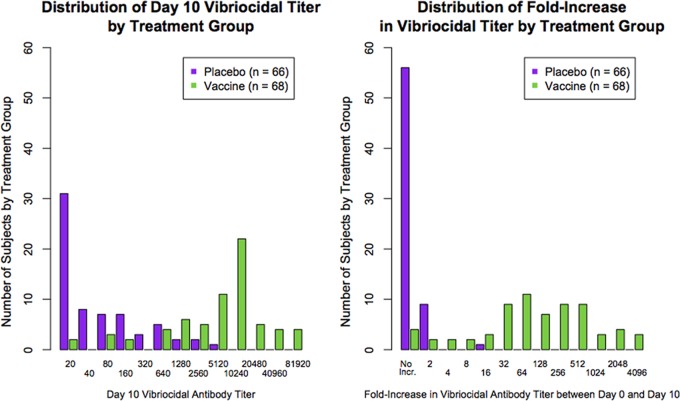
Overlap of absolute vibriocidal antibody titer distribution in vaccinees and placebo recipients and overlap of fold increase in vibriocidal antibody titer.

The distribution of the vibriocidal antibody titer on day 10 for the pool of vaccinees from both challenge cohorts as well as for the combined placebo group is shown in the left-hand panel of Fig. 1, and the distribution of the fold increase in vibriocidal antibody titer from day 0 to day 10 for each treatment group is shown in the right-hand panel of Fig. 1.

A greater degree of overlap between the vaccine and placebo treatment groups was observed for the vibriocidal antibody titer than for the fold increase in titer; that is, relatively high absolute titers appeared in both the placebo and vaccine groups, but 4-fold or greater increases were observed predominantly in vaccine recipients (only 1 of 66 placebo recipients had a 4-fold or greater rise in titer).

The overlap between the day 10 vibriocidal antibody titers in the vaccine and placebo groups has important implications for titer-based bridging. Table 3 depicts treatment-specific outcomes for subgroups defined by different cutoffs for the day 10 titer. These results demonstrate that the prechallenge vibriocidal antibody titer cutoff above which individuals appear to be protected was markedly different in the placebo and vaccine arms. Across the two challenge groups, only 1 out of 61 vaccinated subjects (2%) with day 10 vibriocidal antibody titers greater than or equal to 320 developed moderate or severe diarrhea, while 6 of 13 placebo recipients (46%) with day 10 titers greater than or equal to 320 developed moderate or severe diarrhea. A very similar result was obtained if the maximum prechallenge titer was used in place of the day 10 titer.

**TABLE 3:** Outcomes for vibriocidal antibody titer and fold increase cutoffs by treatment group

Assessment	% subjects who developed moderate/severe diarrhea (no. of subjects who met the specified criterion/total no. tested in each group)	
	Vaccine group	Placebo group
Vibriocidal antibody titer on day 10		
≥20	9 (6/68)	59 (39/66)
≥40	8 (5/66)	54 (19/35)
≥80	8 (5/66)	52 (14/27)
≥160	3 (2/63)	45 (9/20)
≥320	2 (1/61)	46 (6/13)
≥640	2 (1/61)	50 (5/10)
≥1,280	0 (0/57)	40 (2/5)
≥2,560	0 (0/51)	33 (1/3)
Fold increase in vibriocidal antibody titer between day 0 and day 10		
>0	9 (6/68)	59 (39/66)
≥2	5 (3/64)	70 (7/10)
≥4	3 (2/62)	0 (0/1)
≥8	2 (1/60)	0 (0/1)
≥16	0 (0/58)	0 (0/1)
≥32	0 (0/55)	0 (0/0)
≥64	0 (0/46)	0 (0/0)

The existence of atypically high vibriocidal antibody titers in some placebo recipients and the marginal improvement in outcomes for these subjects indicate that factors unrelated to vaccination and only weakly related to protection can cause vibriocidal antibody titers to be elevated. These noise factors would decrease the statistical power of bridging procedures that rely upon the absolute titer to evaluate the equivalence of the immune response to vaccine in different populations.

Table 3 depicts the treatment-specific outcomes for subgroups defined by different cutoffs for the fold increase in vibriocidal antibody titer from day 0 to day 10. These results show that substantial fold increases in titer occur almost exclusively in vaccinees, and thus, members of a bridging population will have a sizable fold increase predominantly as a result of vaccination and not because of weakly protective factors that are independent of vaccination.

### Bridging statistics based upon vibriocidal correlates of protection.

Two-by-two tests revealed significant associations between the fold increase in vibriocidal antibody titer in vaccinees and the clinical outcome for several levels of fold increase ranging from 4- to 32-fold (see Table SA1 in the supplemental material). While any of these fold increase levels could reasonably function as a cutoff to predict whether a vaccinee would be protected from infection, the intended use of the cutoff as a bridging statistic helped guide the selection of a particular cutoff for further evaluation. Since bridging allows efficacy to be inferred across populations, a useful bridging cutoff should identify vaccinees who have a better chance of being protected than placebo recipients. A cutoff that indicates perfect protection is not required since it might exclude a group of subjects that was partially protected by the vaccine. Accordingly, a 4-fold cutoff was selected since nearly all vaccinees who were protected (60/62, 97%) had a 4-fold or greater increase in vibriocidal antibody titer. A secondary advantage of using a 4-fold cutoff is that seroconversion for titer assays is classically defined as a 4-fold or greater increase from the prevaccination titer (10); thus, the cutoff explored below adheres to an existing framework for defining seroconversion.

### Performance of vibriocidal antibody seroconversion.

The rate of vibriocidal antibody seroconversion prior to challenge was 94% for vaccinees in the group challenged at 10 days and 88% for vaccinees in the group challenged at 90 days (Table 4), and moderate/severe diarrhea was observed in just under 10% of vaccine recipients: 6% (2/35) in the cohort challenged at 10 days and 12% (4/33) in the cohort challenged at 90 days. Conversely, only 1 (2%) placebo recipient seroconverted (Table 4) and 59% (39/66) of placebo recipients developed moderate/severe diarrhea. Ninety-seven percent of vaccinees who seroconverted were protected (Table 5).

**TABLE 4:** Proportions of vibriocidal seroconverters at day 10 by treatment group

Group	No. of subjects		% seroconverters	95% CI on proportion of seroconverters
	Randomized	Seroconverters at day 10		
Vaccine				
Challenged at day 10	35	33	94	(81, 99)
Challenged at day 90	33	29	88	(72, 97)
Placebo, challenged on day 10 or day 90	66	1	2	(0, 8)

**TABLE 5:** Rates of protection against moderate/severe diarrhea in seroconverting vaccinees

Day of challenge	No. of subjects		Rate (%) of protection among seroconverters	95% CI on rate of protection
	Vibriocidal seroconverters at day 10	Seroconverters who were protected against moderate/severe diarrhea		
10	33	32	97	(84, 100)
90	29	28	97	(82, 100)

### Response-based prediction of outcome in individual vaccinees.

The tight link between the vibriocidal response to the vaccine and outcome makes it possible to form predictions about protection against controlled exposure to V. cholerae for strong responders to the vaccine (Table 6). For example, among 58 subjects with a vaccine-induced fold increase in vibriocidal antibody titer that was greater than or equal to 16, 0 developed moderate/severe diarrhea, and thus, 100% of the robust responders were protected against cholera challenge (95% confidence interval [CI], 94% to 100%). On the other hand, there were relatively few weak responders to the vaccine, and thus, substantial uncertainty regarding the protectiveness of the vaccine in such cases makes predictive statements inadvisable.

**TABLE 6:** Attack rate of moderate/severe diarrhea versus day 10 vibriocidal antibody titer and fold increase in vibriocidal antibody titer from day 0 to day 10

Assessment	No. of vaccinees	Attack rate (%)	95% CI on attack rate
Day 10 vibriocidal antibody titer			
20–80	5	80	(28, 99)
160–640	6	33	(4, 78)
1,280+	57	0	(0, 6)
Fold increase from day 0 to day 10			
≤2	6	67	(22, 96)
4–8	4	50	(7, 93)
16+	58	0	(0, 6)

### Anti-CT antibodies as a potential correlate of protection.

As with vibriocidal antibodies, the maximum anti-CT antibody response was presumed to offer the most promise as a potential correlate of protection. However, vaccinees did not attain their maximum anti-CT antibody GMT until day 28 (see the supplemental material), which was too late to be observed in the cohort that was challenged at day 10. Thus, the following analysis of the relationship between the anti-CT response and the total postchallenge stool volume relied only on data from the group challenged at 90 days.

### Association between anti-CT response and outcome.

There was no detectable association between the day 28 anti-CT antibody titer and the postchallenge stool volume (*r* = −0.06, *P* = 0.74, *n* = 33) or between the postvaccination fold increase in anti-CT antibody titer and the postchallenge stool volume (*r* = −0.16, *P* = 0.36, *n* = 33) for vaccinees in the group challenged at 90 days (Table 7). However, 3 of the 4 vaccinees in this group who developed cholera were among the weakest responders to the vaccine, showing fold increases in anti-CT antibody titer of 1.5 or less, whereas the median fold increase was 10 for protected vaccinees.

**TABLE 7:** Anti-CT antibody titer versus postchallenge stool volume and fold increase in anti-CT antibody titer versus postchallenge stool volume by treatment in the group challenged at 90 days

Assessment	Vaccine group		Placebo group	
	No. of subjects	Mean (median) total stool volume (liters)	No. of subjects	Mean (median) total stool volume (liters)
Day 28 anti-CT antibody titer				
10–399	6	0.8 (0.3)	23	4.9 (4.5)
400–1,599	11	2.1 (0.2)	6	7.7 (5.3)
1,600–6,399	8	0.3 (0.2)	2	4.1 (4.1)
6,400+	8	0.8 (0.5)	2	5.9 (5.9)
Fold increase in anti-CT antibody titer from day 0 to day 28				
≤3.9	10	2.5 (0.3)	32	5.2 (4.6)
4–7.9	4	0.5 (0.3)	1	11.2 (11.2)
8–15.9	9	0.7 (0.2)		
16+	10	0.3 (0.2)		

For placebo recipients in the group challenged at 90 days, there was no association between the postchallenge stool volume and the anti-CT antibody titer on day 28 (*r* = 0.04, *P* = 0.83, *n* = 33). Since anti-CT antibody titers for placebo subjects remained flat over time (see the supplemental material), the association between the fold increase and outcome could not be assessed.

## DISCUSSION

We found that both vaccine-induced vibriocidal antibody seroconversion prior to challenge and a high postvaccination, prechallenge vibriocidal antibody titer were strongly associated with protection against moderate/severe diarrhea among cholera-naive subjects in an experimental challenge trial. As an immunologic correlate of protection for use in bridging studies, vibriocidal antibody seroconversion 10 days after vaccination was better than the absolute day 10 vibriocidal antibody titer.

The advantage of seroconversion over absolute titer as a bridging correlate is a consequence of the presence of high baseline vibriocidal antibody titers in some placebo subjects and the marginal improvement in outcomes for these subjects with high vibriocidal antibody titers. This vibriocidal activity, found in a population that had not been exposed to cholera, suggests that factors unrelated to vaccination or disease exposure and only weakly related to protection may cause vibriocidal antibody titers to be elevated. Explanations for titers that are well above average among unvaccinated, cholera-naive subjects are at present hypothetical. It is possible that this detection of vibriocidal activity reflects exposure to other vibrios (11) or infectious agents that induced cross-reacting vibriocidal antibody levels. Such antibodies might be only weakly protective, or they might not be associated with protection at all. For example, these antibodies might be bactericidal only in an *in vitro* laboratory assay and/or might be bactericidal against V. cholerae but present only in the blood and not at the site of V. cholerae infection in the intestinal lumen. In contrast, the results presented in this paper imply that the increase in vibriocidal antibody titer following vaccination is a proxy for protective, V. cholerae-specific intestinal immune responses, such as the induction of luminal vibriocidal antibodies as well as other humoral and cellular immune factors.

When establishing a framework for using the immunologic response to infer vaccine efficacy in populations of cholera-naive individuals who are planning to travel to areas where cholera is endemic, such as children or the elderly, the shortcomings of the absolute titer extend to other possible titer-based, single-time-point bridging statistics, such as the geometric mean titer (GMT) of vibriocidal antibodies. Since the GMT is a population-level statistic, it does not facilitate assessments of an association with outcome for individual subjects. Furthermore, the GMT shares the limitation of the titer cutoff approach, in that it is based only upon postbaseline titers, with no adjustment for prevaccination antibody levels.

The construct described here for inferring the efficacy of CVD 103-HgR in cholera-naive populations based upon immunologic responses to the vaccine allowed all vibriocidal antibody titers to be assessed at a single laboratory. However, if the vibriocidal response were to be used to assess protection for individual recipients of the vaccine at numerous travel clinics in a region where cholera is not endemic, vibriocidal antibody seroconversion would have an additional potential advantage over the absolute titer due to the fact that variations in titers across laboratories can make it difficult to establish useful protective cutoffs but are likely to have far less impact upon vaccine-induced changes in titer, such as seroconversion, since laboratory-related effects will tend to be similar at the pre- and postvaccination time points for a given individual.

The relationship between vibriocidal antibody titer and protection against moderate/severe diarrhea appears to be different between cholera-naive individuals with above-average titers and those who have been exposed to vibrios or who have received a cholera vaccine. Mosley (4) demonstrated that in East Pakistan (now Bangladesh), an area where cholera is endemic, the vibriocidal antibody titer increased with age and was associated with a pronounced age-related fall in the frequency of cholera cases. In such regions where cholera is endemic, elevated vibriocidal antibody titers likely represent a previous exposure(s) to live cholera organisms. Thus, these antibodies represent potentially protective immune responses induced by these live organisms. The immune response to live organisms is similar to, though likely of greater magnitude than, the responses induced by a single dose of the live, attenuated cholera organism in the CVD 103-HgR vaccine. Our findings in a cholera-naive population indicate that previously unexposed placebo recipients with above-average vibriocidal antibody titers had limited protection against a cholera challenge and that among subjects with similar prechallenge titers, vaccinees were protected far more often than placebo recipients. The differential implications of high vibriocidal antibody titers in different populations suggest the importance of an individual's history of exposure when describing the relationship between vibriocidal antibody titer and protection against cholera.

A case could be made that due to the small proportion of subjects with postvaccination fold increases in vibriocidal antibody titer of 4, 8, or 16, these potential cutoffs are indistinguishable and the selected bridging statistic could have been based upon an 8-fold or 16-fold increase rather than a 4-fold increase. However, the low frequency of these moderate responders also implies that when bridging across populations, the proportion of subjects with at least a 4-fold increase will in fact be similar to the proportion with at least an 8-fold or a 16-fold increase. Thus, uncertainty in the selection of the cutoff for the correlate should have a minimal impact upon bridging for populations with robust responses to the vaccine.

In addition to the historical recognition of the value of the serum vibriocidal antibody titer as a predictor of outcome, the results of a very small study suggested that the anti-CT antibody titer following V. cholerae infection might be associated with protection against subsequent exposure to cholera (12). In this study, protected subjects had both vibriocidal and anti-CT antibodies, so the independent role of the two types of antibodies could not be discerned. A study in an area where cholera is endemic demonstrated that serum vibriocidal and IgA anti-CT antibody levels (but not IgG anti-CT antibody levels) correlated with protection for household contacts of cholera cases (13). While the use of multiple correlates of protection to infer efficacy in unchallenged populations is worthy of consideration, the nearly one-to-one relationship between seroconversion of the vibriocidal antibody titer and protection in the current study indicates that utilizing a second correlate in combination with seroconversion would have minimal value at best. A second predictor of protection would be useful if some vaccinees seroconverted but were not protected or failed to seroconvert but were still protected. Both of these scenarios were rare in the two challenge cohorts, with one subject of each type being found in each cohort. Future clinical studies with larger sample sizes might provide sufficient information to identify one or more cocorrelates, in addition to vibriocidal antibody seroconversion, such as serum IgA response. For the data set described in this paper, the small number of mismatches between the seroconversion correlate and outcome allowed little room for improvement through the addition of a cocorrelate, such as the fold increase in anti-CT antibody titer.

There is still a clinical need for correlates of protection against cholera that support decision-making with regard to individuals who are at risk, and research into correlates for recipients of CVD 103-HgR is ongoing. Samples from the challenge study described here are being used to assess antibodies against the O-specific polysaccharide component of LPS as possible correlates of protection. In addition, anti-LPS and anti-CT memory B cells in the blood are being evaluated as potential correlates of long-term protection (5). These memory B cells presumably have the capacity to differentiate into antibody-secreting cells (ASCs) and might contribute to an anamnestic response if they are still present at the time of reexposure. It has already been shown that antigen-specific memory B cells can be detected in the blood for at least 1 year after acute infection with wild-type V. cholerae strains (14).

It is important to note that the relationship between vibriocidal antibody seroconversion and protection against cholera might well be different for live and killed cholera vaccines. Since the vibriocidal response is a nonmechanistic correlate of protection, it is possible that some of the protective processes that are reflected by seroconversion could be vaccine specific. It has been hypothesized that one of the advantages of live attenuated bacterial vaccines is the potential to express and then stimulate responses to antigens that are expressed only during the course of infection and are therefore not present in killed vaccines. For example, in a subset of the participants in the challenge trial described here, CVD 103-HgR primed a response to the toxin-coregulated pilus antigen TcpA that was observed subsequent to the challenge at 90 days but not after the challenge at 10 days, suggesting that the priming was dependent upon the development of a memory B cell response to the live vaccine that took over 10 days to develop (15). Further studies are required to compare the immunologic memory processes induced by live and killed cholera vaccines and to assess the value of potential correlates of protection for different types of cholera vaccines.

One limitation of this study is that the challenge model in cholera-naive individuals may have limited implications for populations in settings where cholera is endemic. Still, it is possible that the vibriocidal response correlates with protection in these populations as well, and results from challenge, case-control, and controlled field trials might provide additional insights in these settings. A second limitation relates to the fact that vaccine efficacy was established only at 10 and 90 days postvaccination. While vibriocidal antibody seroconversion was associated with protection at both time points, vaccine efficacy might wane after 90 days, and vibriocidal antibody seroconversion at day 10 might not predict which subjects will remain protected and which subjects will not remain protected at later time points.

In summary, our findings suggest that in a highly controlled clinical trial setting, vibriocidal antibody seroconversion had distinct advantages over the vibriocidal antibody titer at a single time point for the comparison of immune responses to vaccine in different cholera-naive populations. Although both the fold increase in the vibriocidal antibody titer and the actual vibriocidal antibody titer were predictive of protection against cholera challenge in individual subjects, the presence of serum vibriocidal activity in some individuals prior to vaccination implies that the fold increase in the vibriocidal antibody titer provides a greater statistical power than the absolute titer for bridging applications. Thus, in future bridging studies involving cholera-naive populations, our data support the use of a 4-fold or greater increase in the vibriocidal antibody titer as a robust correlate of protection.

## MATERIALS AND METHODS

### Description of cohort.

This study was approved by institutional review boards at the three enrollment centers (Center for Vaccine Development, University of Maryland School of Medicine, Baltimore, MD; Cincinnati Children’s Hospital Medical Center, Cincinnati, OH; and Vaccine Testing Center, University of Vermont College of Medicine, Burlington, VT). The analysis population comprised 68 vaccinated subjects and 66 placebo recipients who were challenged at either 10 or 90 days postvaccination. The design and outcome of the study have been described previously (1). Briefly, study volunteers were randomized to receive CVD 103-HgR or placebo in a 1:1 ratio. Vaccine and placebo recipients were then challenged with virulent V. cholerae O1 El Tor Inaba, a biotype that is heterologous to the classical Inaba strain used in the vaccine, at either 10 days or 90 days postvaccination.

### Measurement of immune responses.

Prechallenge serum vibriocidal antibody titers for the 35 vaccinees and 33 placebo recipients in the trial in which the subjects were challenged at 10 days were assessed on days 0, 7, and 10, and prechallenge titers for the 33 vaccinees and 33 placebo recipients in the trial in which the subjects were challenged at 90 days were assessed on days 0, 7, 10, 28, and 90. The cohorts for the two challenge trials were unique and nonoverlapping. Blood samples for measurements of serum antibody levels on day 0 were taken prior to the administration of vaccine or placebo. Antibody titers for samples that were drawn just prior to the administration of the cholera challenge at 10 days in the first cohort are referred to as day 10 results, and antibody data collected just prior to the administration of the challenge in the cohort challenged at 90 days are referred to as day 90 results.

Titers of vibriocidal antibodies against the classical Inaba cholera strain were assessed at Focus Diagnostics, Inc., using an assay that was transferred from the Center for Vaccine Development at the University of Maryland (CVD-UMB). The vibriocidal antibody assay (16) was performed by diluting serum from 1:10 to 1:10,240, mixing the diluted serum with an equal volume of standardized V. cholerae bacteria (including guinea pig complement) for final dilutions of 1:20 to 1:20,480, and incubating the mixture for 1 h at 37°C. After incubation, an additional volume of brain heart infusion broth was added and the plates were incubated for approximately 2 to 3 h at 37°C. Titers were reported as the reciprocal of the dilution of the most diluted sample with an optical density (measured at 620 nm) that was associated with at least a 75% reduction in bacterial growth.

The titers of serum IgG antibodies that bound to the B subunit of cholera toxin were quantitated using an enzyme-linked immunosorbent assay that was transferred from CVD-UMB to Focus Diagnostics, Inc. The assay was performed by serial dilution of samples from 1:200 to 1:12,800 in duplicate, incubation on CT-coated wells, and detection with peroxidase-labeled goat anti-human IgG antibodies and a tetramethylbenzidine (TMB) substrate. The plates were read at 450 nm. For each subject, the mean of the two background-corrected optical densities at each dilution level was calculated. A continuous relationship between the mean optical density and the log_2_ reciprocal dilution was interpolated using fourth-order polynomial regression. The titer was defined to be the greatest value of the reciprocal dilution that has a corresponding fitted optical density greater than or equal to 0.2.

### Endpoints.

The primary efficacy endpoint in the challenge trial was moderate to severe diarrhea, defined as a ≥3-liter total volume of diarrhea postchallenge, and this study used the same endpoint to evaluate potential correlates of protection. The primary endpoint was chosen because it represents a clinically severe manifestation of disease and has been a component of the CVD volunteer model of experimental cholera that was established in 1976 in collaboration with the National Institutes of Health. Antibiotics were administered if a subject developed severe diarrhea (total stool volume, at least 5 liters) or at 4 days after challenge, whichever came first. The proportion of subjects in a treatment group who met the criteria for moderate/severe diarrhea is referred to as the attack rate. Vaccinees are said to have been protected if they did not meet the criteria for moderate or severe diarrhea. Since CVD 103-HgR was quite effective in preventing moderate to severe diarrhea in the challenge trial, the trial data set is suboptimal for the assessment of correlates of protection, since it is difficult to evaluate a potential correlate without data from substantial numbers of both protected and unprotected vaccinees. In order to add resolution to the outcome data and thereby increase the statistical power, the total postchallenge stool volume as a continuous value was used as a second endpoint for the evaluation of correlates.

### Statistical methods.

The geometric mean titers (GMTs) of vibriocidal antibodies against the classical Inaba strain and of anti-CT antibodies were used to describe the longitudinal profile of the immune response to the vaccine. Evaluation of trends in total stool volume as a function of the postvaccination, prechallenge vibriocidal antibody titer or the fold increase in titer were performed using the nonparametric Jonckheere-Terpstra trend test (17). Seroconversion of vibriocidal antibodies or anti-CT antibodies was considered to be cumulative and was defined at a given point in time as a 4-fold or greater increase in titer from the pretreatment titer at that time or at any preceding posttreatment assessment. The association between the total stool volume and continuous anti-CT antibody titer or the fold increase in the anti-CT antibody titer was summarized using Spearman's rank correlation (18).

To identify potential bridging methods based upon the postvaccination, prechallenge vibriocidal response, a series of cutoffs was selected, and for each cutoff, a binary variable was created by assigning all vaccinees with responses greater than or equal to the cutoff to one group and the remaining vaccinees to a second group. The strength of the relationship between each of these cutoff-specific binary variables and outcome was assessed by conducting a two-by-two chi-square test of association with the occurrence of moderate/severe diarrhea as the second binary factor.

Confidence intervals for proportions were computed using the Clopper-Pearson method (19).

## Supplementary Material

Supplemental material
