# Supplementary material for: Antibody-Based Correlates of Protection against Cholera: Analysis of a Challenge Study of a Cholera-Naive Population
Source: Clin Vaccine Immunol. 2017 Aug 4;24(8):e00098-17. doi: 10.1128/CVI.00098-17 (PMC5583470; doi:10.1128/CVI.00098-17)
Supplement: Supplemental material [file supp_24_8_e00098-17__index.html]

Supplemental material 

# Antibody-Based Correlates of Protection against Cholera: Analysis of a Challenge Study of a Cholera-Naive Population

## Supplemental material

- Supplemental file 1 -

  Fig. SA1. Distribution of vibriocidal antibody titers on day 1 for placebo and vaccine groups. Table SA1. Fold increase in vibriocidal titer in vaccinees and clinical outcome for several levels of fold increase ranging from 4- to 32-fold. Table SA2. Several titer cutoffs and fold-increase cutoffs produce associations with outcome that are of comparable strength. Table SA3. Anti-CT GMTs over time. Table SA4. Proportions of anti-CT seroconverters in 10- or 90-day challenge by treatment group.

  PDF, 284K
