# Supplementary material for: Antibody-Based Correlates of Protection against Cholera: Analysis of a Challenge Study of a Cholera-Naive Population
Source: Clin Vaccine Immunol. 2017 Aug 4;24(8):e00098-17. doi: 10.1128/CVI.00098-17 (PMC5583470; doi:10.1128/CVI.00098-17)
Supplement: Supplemental material [file CVI.00098-17_zcd999095499s1.pdf]

Appendix

Figure A1
Distribution of Vibriocidal Antibody Titer on Day 1 for Placebo and Vaccine Groups

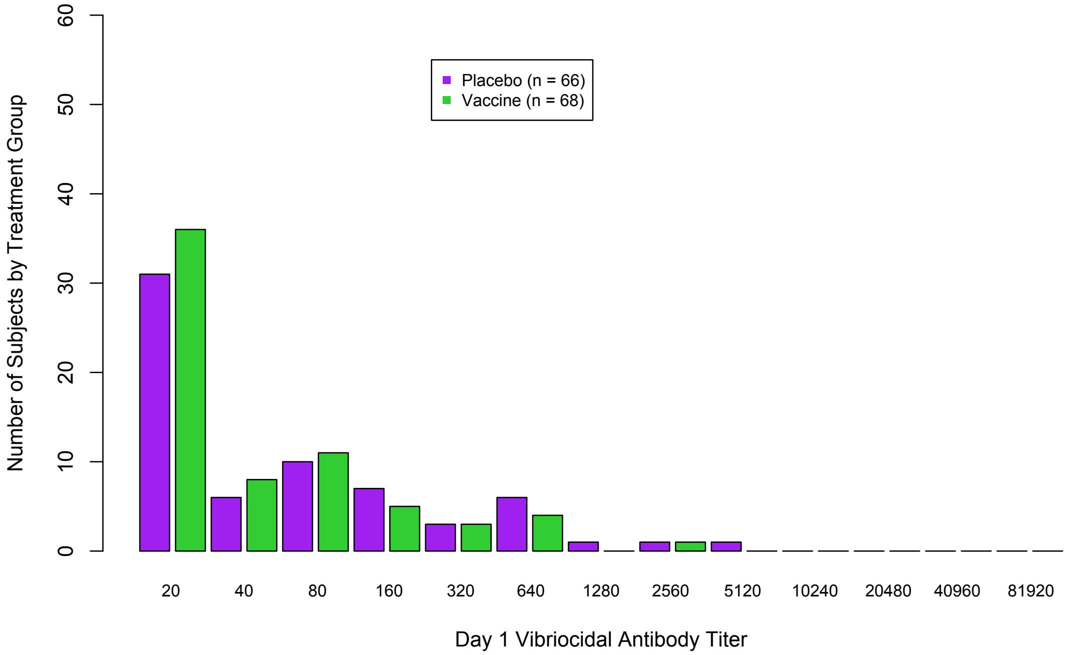

Table A1

Measure of Immune Response	Cutoff	Chi-square	p-value
Day 10 Vibriocidal Titer	160	25	< 0.001
Day 10 Vibriocidal Titer	320	30	< 0.001
Day 10 Vibriocidal Titer	640	30	< 0.001
Day 10 Vibriocidal Titer	1280	28	< 0.001
Fold-Increase in Vibr. Titer from Day 0 to Day 10	4	20	< 0.001
Fold-Increase in Vibr. Titer from Day 0 to Day 10	8	25	< 0.001
Fold-Increase in Vibr. Titer from Day 0 to Day 10	16	31	< 0.001
Fold-Increase in Vibr. Titer from Day 0 to Day 10	32	22	< 0.001

Inspection of the tables in Table A2 reveals that several titer cutoffs and fold-increase cutoffs produce associations with outcome that are of comparable strength. For example, titer cutoffs from 160 to 1280 all produce 2 x 2 chi-square values greater than 20 and p-values less than 0.001, as do fold-increase cutoffs from 4 to 32.

The cutoffs associated with the Day 10 vibriocidal titer that produced the maximum observed chi-square statistic were 320 and 640 (chi-square = 30, $p < 0.001$), and the cutoff on the fold-increase in titer that produced the maximum chi-square statistic was 16 (chi-square = 31, $p < 0.001$).

Table A2a

		Moderate/Severe Cholera	
		Yes	No
Day 10 Vibriocidal Titer >= 320	Yes	1	60
	No	5	2

Table A2b

		Moderate/Severe Cholera	
		Yes	No
Day 0 to Day 10 Fold-Increase in Vibriocidal Titer >= 16	Yes	0	58
	No	6	4

Anti-CT GMTs over Time

Tables A3a and A3b contain the geometric mean of the anti-CT titer over time for each treatment, split by challenge group. The post-vaccination geometric mean titer of anti-CT antibodies reached a maximum at Day 28, too late to be observed in the 10-day challenge group. Thus, titers from the 10-day and 90-day challenge groups were not pooled, and subsequent analysis of the relationship between anti-CT response and total stool volume focused on the data from the 90-day group. Anti-CT titers for placebo subjects were flat over time.

Table A3a

Pre-challenge Anti-CT GMTs by Timepoint
for Vaccinees in Each Challenge Group

	Vaccinees Challenged at Day 10			Vaccinees Challenged at Day 90	
	Unprotected	Protected		Unprotected	Protected
n	2	33		4	29
Day 0 GMT	259	227		347	179
Day 7 GMT	259	334		675	454
Day 10 GMT	291	770		997	1218
Day 28 GMT	-----	-----		827	2056
Day 90 GMT	-----	-----		572	1661

Table A3b

Pre-challenge Anti-CT GMTs by Timepoint
for Placebo Subjects in Each Challenge Group

	Placebo Subjects Challenged at Day 10			Placebo Subjects Challenged at Day 90	
	Developed Moderate/Severe Cholera	Did Not Develop Moderate/Severe Cholera		Developed Moderate/Severe Cholera	Did Not Develop Moderate/Severe Cholera
N	20	13		19	14
Day 0 GMT	298	282		252	257
Day 7 GMT	320	190		253	263
Day 10 GMT	296	223		265	250
Day 28 GMT	-----	-----		234	283*
Day 90 GMT	-----	-----		237	217

*Anti-CT data for one placebo subject in the 90-day challenge group were not available on Day 28.

Table A4a shows the proportion of subjects in the 10-day challenge study who experienced a four-fold or greater increase in anti-CT titer between Day 0 and Day 10 in each treatment group, and Table A4b indicates the proportion of subjects in the 90-day study who experienced a four-fold or greater increase in anti-CT titer between Day 0 and Day 28 by treatment group. As stated earlier, the post-vaccination anti-CT titer was still increasing at the time of the 10-day challenge, so it is difficult to identify all vaccine responders in the 10-day challenge group. In the 90-day challenge group, 73% of vaccinees experienced a four-fold or greater increase in anti-CT titer by Day 28, when the GMT reached its peak.

Table A4a

Proportions of Anti-CT Seroconverters in 10-Day Challenge by Treatment Group

	Randomized	Seroconverters at Day 10	Proportion of Seroconverters	95% CI on Proportion of Seroconverters
Vaccine	35	13	37%	(21%, 55%)
Placebo	33	1	3%	(0.1%, 16%)

Table A4b

Proportions of Anti-CT Seroconverters in 90-Day Challenge by Treatment Group

	Randomized	Seroconverters at Day 28	Proportion of Seroconverters	95% CI on Proportion of Seroconverters
Vaccine	33	24	73%	(54%, 87%)
Placebo	33	1	3%	(0.1%, 16%)

*One vaccinee who seroconverted prior to Day 28 had an anti-CT titer on Day 28 that was not at least 4-fold greater than the Day 0 titer. This subject is considered to be a seroconverter on Day 28 using the cumulative definition of seroconversion.
